# Two Coiled-Coil Domains of *Chlamydia trachomatis* IncA Affect Membrane Fusion Events during Infection

**DOI:** 10.1371/journal.pone.0069769

**Published:** 2013-07-23

**Authors:** Erik Ronzone, Fabienne Paumet

**Affiliations:** Department of Microbiology and Immunology, Thomas Jefferson University, Philadelphia, Pennsylvania, United States of America; Medical College of Georgia, United States of America

## Abstract

*Chlamydia trachomatis* replicates in a parasitophorous membrane-bound compartment called an *inclusion*. The inclusions corrupt host vesicle trafficking networks to avoid the degradative endolysosomal pathway but promote fusion with each other in order to sustain higher bacterial loads in a process known as *homotypic fusion*. The *Chlamydia* protein IncA (Inclusion protein A) appears to play central roles in both these processes as it participates to homotypic fusion and inhibits endocytic SNARE-mediated membrane fusion. How IncA selectively inhibits or activates membrane fusion remains poorly understood. In this study, we analyzed the spatial and molecular determinants of IncA’s fusogenic and inhibitory functions. Using a cell-free membrane fusion assay, we found that inhibition of SNARE-mediated fusion requires IncA to be on the same membrane as the endocytic SNARE proteins. IncA displays two coiled-coil domains showing high homology with SNARE proteins. Domain swap and deletion experiments revealed that although both these domains are capable of independently inhibiting SNARE-mediated fusion, these two coiled-coil domains cooperate in mediating IncA multimerization and homotypic membrane interaction. Our results support the hypothesis that *Chlamydia* employs SNARE-like virulence factors that positively and negatively affect membrane fusion and promote infection.

## Introduction

*Chlamydia* remains a significant socioeconomic and medical burden despite decades of steadfast research. *Chlamydia trachomatis* is the causative agent of chlamydiosis in humans. Infection is associated with pelvic inflammatory disease in women and severe tissue scarring and infertility in both men and women. Inoculation of the conjunctiva with *C. trachomatis* leads to inflammation and trachoma–the leading cause of infectious blindness in the world [1]. In 2011, over 1.4 million cases of *Chlamydia* infection were reported, making *Chlamydia* one of the most frequently reported sexually transmitted diseases in the United States (US Centers for Disease Control).

*Chlamydiae* exhibit a biphasic lifecycle, existing as metabolically inert but infectious elementary bodies (EBs) outside the cell and actively dividing reticulate bodies (RBs) inside. EBs direct their own internalization into host cells–typically mucosal epithelial cells–and modify the nascent phagosome into a replicative niche called an *inclusion* [2]. Between 10 and 12 hours post infection (hpi), two or more inclusions in the same cell begin to fuse into a single large inclusion in a process known as *homotypic fusion* [3]. Strains of *Chlamydia* that do not fuse their inclusions are unable to generate high bacterial loads and are less pathogenic than fusion-competent strains [4], [5]. Unlike phagosomes of non-pathogenic bacteria that ultimately traffic to lysosomes, the inclusion interacts with early (and possibly late) endocytic compartments, but avoids lysosomes altogether [6]–[10]. Early endocytic markers like transferrin receptor cluster around the inclusion ∼4 hours post infection (hpi), but lysosomal markers LAMP-1 and LAMP-2 are not detectable as late as 20 hpi [9], [10]. Importantly, the failure of these markers to appear on the inclusion is not due to a global breakdown of the endocytic process as yeast particles are still trafficked successfully to lysosomes in *Chlamydia*-infected cells [11]. These observations suggest that the avoidance of lysosomes is an active process and is intrinsic to the inclusion.

Canonical phagosomes successively fuse with early and late endosomes before fusing with lysosomes in a tightly regulated manner [12]. At the heart of this process are SNARE (soluble N-ethylmaleimide sensitive factor attachment receptor) proteins. SNAREs reside on the cytosol-exposed surfaces of membranous organelles and catalyze the specific fusion of intracellular compartments [13]–[15]. The SNAREs controlling late endosome/lysosome fusion are Syntaxin7 (Stx7), Stx8, Vti1b, and VAMP8 [16]–[18]. Stx7, Stx8, and Vti1b form a t-SNARE (target-SNARE) complex on the target membrane. This complex, in turn, binds the cognate vesicle-localized SNARE (v-SNARE) VAMP8 *in trans* to form a four-helix coiled-coil bundle that fuses late endosomes and lysosomes. The centrality of SNARE proteins to trafficking makes them ideal targets for pathogens attempting to establish residence in the cell [19].

Previously, we have demonstrated that IncA, a *Chlamydia* protein that resides on the inclusion membrane, inhibits the fusion of liposomes carrying Stx7, Stx8, Vti1b, and VAMP8 [20]. This inhibition is specific because the rate of fusion of liposomes reconstituted with the exocytic SNARE complex Stx4, SNAP23, and VAMP8 is not affected [19], [20]. Interestingly, IncA has also been implicated in homotypic fusion of the inclusions [3]. How IncA performs these two functions is unknown.

IncA contains a transmembrane domain (TMD) flanked by two cytosol-exposed regions on either side [21]. We will refer to these domains as the N-terminal tail and the C-terminal cytoplasmic domain. The C-terminal cytoplasmic domain itself contains two putative coiled-coil domains (CCDs) that show strong homology to eukaryotic SNARE motifs [20]. Interestingly, mutations in IncA’s CCDs ablated binding to VAMP8 *in vivo* [22]. However, the role of the N-terminal tail, as well as the function of each individual SNARE-like motif in the C-terminal cytoplasmic domain, remains unclear.

In this report, IncA’s proper topological organization to exert its function was first determined. Using this topology, we next determined the role of each protein domain (i) for IncA to inhibit SNARE-mediated membrane fusion and (ii) for IncA to interact with itself and induce homotypic fusion. We found that optimal SNARE-mediated membrane fusion inhibition requires that IncA be on the same membrane as the SNARE proteins. A series of mutations and truncations revealed a previously unknown role for the C-terminal coiled-coil domain (CCD) of IncA as an independent inhibitor of membrane fusion. However, *in vivo* characterization of these IncA mutants uncovered that cooperation between the two CCDs is necessary to mediate homotypic fusion.

Altogether, our data identify two functional SNARE-like domains (SLDs) in IncA. We show for the first time that the N-terminal SNARE-like domain SLD1 and the C-terminal SNARE-like domain SLD2 can work independently to inhibit membrane fusion. However, they are both required to promote homotypic fusion. Our data provide new insight regarding how *C. trachomatis* interferes with membrane fusion and support the hypothesis that intracellular pathogens utilize SNARE-like proteins to promote infection.

## Experimental Procedures

### DNA Constructs

A list of primers and sequences are summarized in Table S1. The plasmid encoding 6xHis-Δ34IncA (FD231) was generated by PCR using primers FO136 and FO137 and plasmid FD201 (expression vector for IncA-6xHis wildtype) as template. The resulting PCR product was digested with EcoRI and BamHI (New England Biolabs, NEB) and ligated into pET28a (Novagen).

The expression plasmid encoding 6xHis-ΔTMD-IncA (FD199) and 6xHis-TfR-IncA (FD204) were a kind gift from Dr. A. Subtil. To create the expression plasmid for GST (glutathione S-transferase)-TfR-IncA, the coding sequence for TfR-IncA was cleaved from plasmid FD204 with NdeI and EcoRI and ligated into pGEX vector [23]. Site-directed mutagenesis of truncated (IncA_1–141_) and full-length IncA was accomplished using the QuickChange system (Stratagene). Mutagenic primers FO401 and FO402 were used to introduce the mutations I106D/T127D/V134D into plasmids FD229 (expression plasmid for 6xHis-IncA_1–141_) and FD201 to generate plasmids FD465 (encoding 6xHis-Asp-IncA_1–141_) and FD464 (encoding Asp-IncA-6xHis), respectively. Mutagenic primers FO414 and FO416 were used to introduce mutations F108A/F117A/F124A/F138A into FD465 to generate FD475 (encoding 6xHis-Phe/Asp-IncA_-141_). Primers FO414 and FO415 were used to introduce mutations F108A/F117A/F124A/F138A/F145A into FD464 to generate FD472 (encoding Phe/Asp-IncA-6xHis). GST was fused to the C-terminal cytoplasmic domains of IncA_1–141,_ Phe/Asp-IncA_1–141_, and Phe/Asp-IncA by first amplifying the gene segment encoding the cytoplasmic domain using primers FO399 and FO400 (for GST-IncA_1–141_ and GST-Phe/Asp-IncA_1–141_) and FO399 and FO137 for GST-Phe/Asp-IncA and ligating into pGEX vector.

pDsRed-IncA plasmids were constructed by amplifying the open reading frames of FD201, FD229, FD231, FD472, and FD475 using primers FO124 and FO125 (for wildtype and Phe/Asp-IncA), FO124 and FO162 (for IncA_1–141_ and Phe/Asp-IncA_1–141_), or FO441 and FO125 for Δ34-IncA and ligating into the pDs-Red-monomer-C1 vector (kindly provided by Dr. P. Antinozzi). All constructs were verified by sequencing at the Nucleic Acids Facility at Thomas Jefferson University.

### Protein Induction and Purification

BL21(DE3) carrying the plasmids of interest were grown in Luria-Bertani (LB) liquid medium supplemented with appropriate antibiotic (50 µg/mL kanamycin and/or 100 µg/mL ampicillin) at 37°C to mid-log phase before adding isopropyl-β-D-1-thiogalactopyranoside to a final concentration of 0.2 mM. Incubation temperature was then lowered to 16°C and the culture was incubated overnight. Induced BL21(DE3) were collected by centrifugation and the pellets stored at −20°C.

For protein purification, bacterial pellets were resuspended in Breaking Buffer (200 mM KCl, 25 mM HEPES, 10% glycerol, 4% Triton X-100, pH7.4) supplemented with 5 mM β-mercaptoethanol and protease inhibitors. Bacteria were lysed by passage through an EmulsiFlex-C3 homogenizer (Avestin). Cellular debris was pelleted by ultracentrifugation. To purify 6xHis-tagged proteins, supernatants containing tagged proteins were incubated with nickel nitriloacetic acid (Ni-NTA) agarose beads (Qiagen) for 1 h at 4°C. Beads were washed with 30–100 mM imidazole in Buffer A (200 mM KCl, 25 mM HEPES, 10% glycerol, 0.8% n-octyl-β-D-glucopyranoside, pH7.4 with 5 mM β-mercaptoethanol). Proteins were eluted with Buffer A containing 500 mM imidazole. For GST-tagged protein purification, supernatants containing the GST-proteins were incubated with glutathione agarose beads (Sigma Aldrich) for 1 h at 4°C. Beads were collected on a Poly-Prep column and washed with three column volumes (∼30 mLs) of Buffer A. Proteins were cleaved from the GST tag and eluted from the agarose matrix using thrombin. Briefly, thrombin (Fisher Scientific) was diluted to 0.05 U/ul in Buffer A and incubated with the agarose matrix for 2 h at room temperature. Thrombin reactions were stopped by adding 4-(2-aminoethyl) benzenesulfonyl fluoride hydrochloride (AEBSF, Fisher Scientific).

### Proteoliposome Reconstitution and Liposome Fusion Assay

Liposomes were prepared as described [24], [25]. The lipid components in the acceptor liposomes are 85% palmitoyl-oleoyl-phosphatidylcholine (POPC) and 15% 1,2-dioleoyl-phosphatidylserine (DOPS), corresponding to 15 mM total lipids in CHCl_3_. The donor liposomes contain 82% POPC, 15% DOPS, 1.5% 7-nitrobenz-2-oxa-1,3-diazole-dipalmitoyl phosphatidyl ethanolamine (NBD-DPPE), 1.5% Rhodamine-DPPE, corresponding to 3 mM total lipids in CHCl_3_. All lipids were obtained from Avanti Polar Lipids, Inc. Briefly, recombinant proteins were mixed with lipids in the presence of detergent for 30 min at room temperature. Dialysis buffer (200 mM KCl, 25 mM HEPES, 10% glycerol, 1 mM dithiothreitol, pH7.4) was added to the mixture to form liposomes, and detergent was removed by overnight dialysis at 4°C. Liposomes were further purified by density gradient centrifugation.

The liposome fusion assay was performed essentially as described [24], [25]. 45 µL of t-SNARE liposomes and 5 µL of v-SNARE liposomes were mixed and incubated at 37°C for 2 h. Nitrobenzoxadiazole (NBD) fluorescence was measured every 2 min. Maximum fluorescence values resulting from complete dequenching of NBD were obtained by adding 10 µL of n-dodecyl-β-D-maltoside detergent (stock concentration 2.5% w/v) to each reaction after the initial 2 h incubation. The percent fluorescence was calculated as follows:
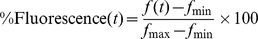
where *f*(*t*) is the fluorescence at time *t*, *f_min_* is the minimum fluorescence value observed for that reaction, and *f_max_* is the maximum fluorescence value.

**Figure 1:**
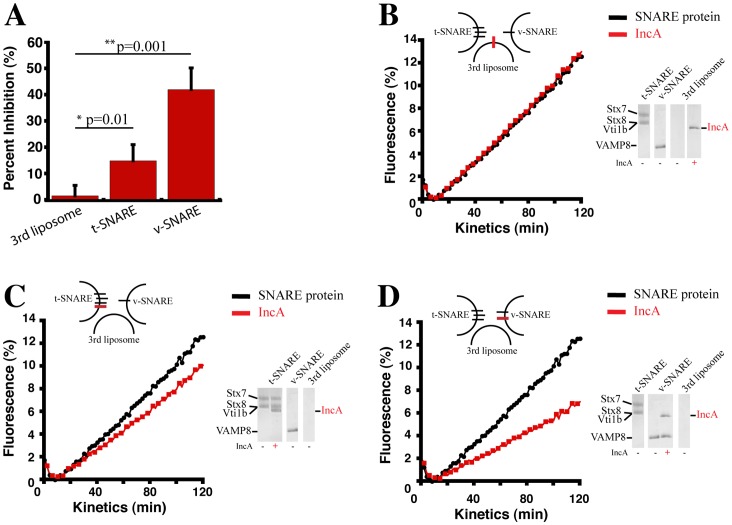
Inhibition of SNARE-mediated fusion by IncA is topologically restricted. The inhibitory capacity of IncA was assessed in three topological configurations–on the t-SNARE liposome, v-SNARE liposome, or a third liposome. 45 µL of t-SNARE liposome reconstituted with Stx7, Stx8, Vti1b were mixed with 5 µL of v-SNARE liposome reconstituted with VAMP8 and 10 µL of third liposome and NBD fluorescence was measured every two minutes for 2 hrs at 37°C. 10 µL of n-Dodecyl-β-D-maltoside (2.5% w/v stock concentration) was added at the end of the 2 hrs and data were normalized to the 100% fluorescence value as described [24]. For each condition, IncA was reconstituted into just one liposome population, and its inhibition tested. (A) The average percent inhibition of IncA in each indicated liposome is presented. Error bars represent one standard deviation from three independent experiments. IncA inhibits SNARE-mediated liposome fusion significantly better when co-reconstituted with SNARE proteins. (B) IncA fails to inhibit when reconstituted singly on the third liposome population. Black circles represent fusion in the absence of IncA, red squares in the presence of IncA. Inset illustrates position of IncA (red bar) relative to SNARE proteins (black bars). SDS-PAGE analysis reveals incorporation of proteins into liposomes. The positions of each protein and liposome population are denoted. The fusion graph is representative of three independent experiments. (C) IncA inhibits fusion when reconstituted with the t-SNAREs. The design of the experiment was identical to (B), except that IncA was reconstituted with the late endocytic t-SNARE complex on the t-SNARE liposome. The fusion graph is representative of three independent experiments. (D) IncA inhibits fusion when reconstituted with the v-SNARE. The design of the experiment was identical to (B), except that IncA was reconstituted with the late endocytic SNARE VAMP8 on the v-SNARE liposome. The fusion graph is representative of three independent experiments.

The topology experiment in Figure 1 followed the same basic protocol above, except that 10 µL of the third liposome population, empty or containing wildtype IncA, was added to each reaction. The percent inhibition by IncA in each configuration was calculated using the equation:
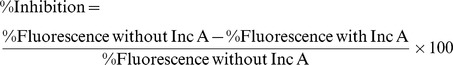



### Circular Dichroism Spectroscopy

ΔTMD-proteins corresponding to the C-terminal cytoplasmic domains of wildtype IncA, IncA_1–141_, Phe/Asp-IncA_1–141_, and Phe/Asp-IncA were GST purified as described above. They were further purified by size-exclusion chromatography using a HiLoad 16/60 pg200 column (GE Lifesciences) equilibrated in 200 mM NaCl, 20 mM Tris, 10% glycerol, pH7.4 supplemented with 5 mM β-mercaptoethanol. Protein-containing fractions were pooled and exchanged into CD Buffer (200 mM NaF, 20 mM Tris, 10% glycerol, pH7.4 with 1 mM dithiothreitol). Protein concentrations were determined by measuring the optical density at 280 nm. Wavelength scans were collected in a 1 mm pathlength quartz cuvette (Starna) using a JASCO J-810 spectropolarimeter equipped with a Peltier temperature control device. Measurements were taken at 20°C.

### Co-elution Assay

BL21(DE3) were co-transformed with plasmid FD439 and either FD201, FD231, FD229, FD475, or FD472. Bacteria were induced overnight as described above. Equal volumes of overnight culture were harvested by centrifugation and resuspended in Buffer A supplemented with 5 mM β-mercaptoethanol and 1 mM AEBSF. Bacteria were sonicated in three 20-second pulses with 1 min recoveries on ice. Cell debris was separated by centrifugation, and the supernatants were incubated over glutathione agarose beads at 4°C for 1 h. Beads were washed twice with Buffer A supplemented with 5 mM β-mercaptoethanol. Bound proteins were eluted by resuspending beads in 2% SDS protein loading buffer before boiling. Equal volumes of each co-elution were run subjected to SDS-PAGE. GST and GST fusion proteins were visualized by Coommassie Blue staining. Co-eluted proteins were visualized by western blot using rabbit α-6xHis primary antibody (Santa Cruz) and donkey α-rabbit horse raddish peroxidase-conjugated secondary antibody (GE Healthcare). Protein bands were detected using enhanced chemiluminescence (ECL) reagents (GE Healthcare). Blots were visualized using a FluorChem M imager (ProteinSimple). As a loading control, 10 µL of supernatant lysate were run on SDS-PAGE and blotted, as above, for 6xHis-tagged proteins.

### Immunofluorescent Microscopy

HeLa cells were cultured in DMEM containing 10% fetal bovine serum, L-glutamine and 10 µg/mL gentamicin. HeLa cells were seeded in 24-well plates at 2.4×10^4^ cells/well and incubated overnight. Cells were transfected using Lipofectamine 2000 (Invitrogen) according to the manufacturer’s protocol. 24 h after transfection, cells were infected with *C. trachomatis*, LGV str. L2 (kindly provided by Dr. T. Hackstadt, NIAID). 24 hpi, cells were fixed in 2% formaldehyde for 30 min at 4°C. DNA was stained with 1 µg/ml Hoechst. Images were acquired using a Nikon Eclipse Ti inverted fluorescence microscope equipped with appropriate filters, 60× oil immersion objective, and NIS Elements software (Nikon). Images were analyzed using ImageJ (NIH).

### Statistical Analysis

Statistics were calculated using Student’s two-tailed t-test. Significance is defined as p≤0.05.

## Results

### Inhibition of SNARE-Mediated Liposome Fusion by IncA is Topologically Restricted

Efficient SNARE-mediated fusion requires that a t-SNARE complex form on one bilayer before interacting with a cognate v-SNARE on an opposing bilayer. This topological restriction ensures aberrant fusion events are avoided and the integrity of organelles is maintained [26]. We theorized that IncA would be similarly topologically restricted. As a result, the inclusion would be protected and fusion with destructive compartments would be prevented. To test this hypothesis, we mimicked different IncA configurations in an *in vitro* fusion assay. We first generated three liposome populations: one carrying the late endocytic t-SNAREs Syntaxin7 (Stx7), Stx8, and Vti1b, one carrying the v-SNARE VAMP8, and a third empty liposome population (Figure 1B, 1C, 1D). Next, we inserted IncA either on the t-SNARE liposome (Fig. 1C), or on the v-SNARE liposome (Fig. 1D), or on the third liposome (Fig. 1B) and calculated the percent inhibition generated by IncA in each topological configuration. The v-SNARE liposomes contain two fluorescent lipids (Rhodamine-phosphatidyl ethanolamine (PE) and NBD-PE), which undergo resonance energy transfer (FRET) when in close proximity such that NBD fluorescence is quenched [25]. Upon membrane fusion with unlabeled t-SNARE liposomes, the fluorophores are diluted and increased NBD fluorescence at 538 nm is measured, providing a quantitative readout of fusion. The third liposome population is also constituted with non-labeled lipids.

Using this setup, we found that IncA requires insertion in the same membrane as the endocytic SNAREs in order to exert its inhibitory function (Fig. 1A). Placing IncA on the third liposome population fails to significantly affect late endocytic SNARE-driven fusion (Figure 1B), and only 1.7% inhibition is observed. SDS-PAGE analysis of liposomes shows incorporation of proteins (Fig. 1B, Coomassie gel). In contrast, the inhibitory capacity of IncA is significantly higher when IncA is reconstituted with either the t- or v-SNARE complex (∼15% and ∼42% inhibition, respectively). As previously demonstrated, the inhibition is less pronounced when IncA is reconstituted on the t-SNARE liposome due to the restricted amount of IncA inserted into these liposomes [20]. Since IncA has been shown to bind VAMP8 *in vitro* and *in vivo*, it is likely that the close proximity of IncA to VAMP8 when both are on the same membrane contributes to the robust inhibition [22]. These data suggest that the topological arrangement of IncA relative to SNARE complexes constitutes a significant factor in its ability to inhibit SNARE complexes.

### The Inhibitory Ability of IncA Is Isolated To The C-terminal Cytoplasmic Domain

**Figure 2:**
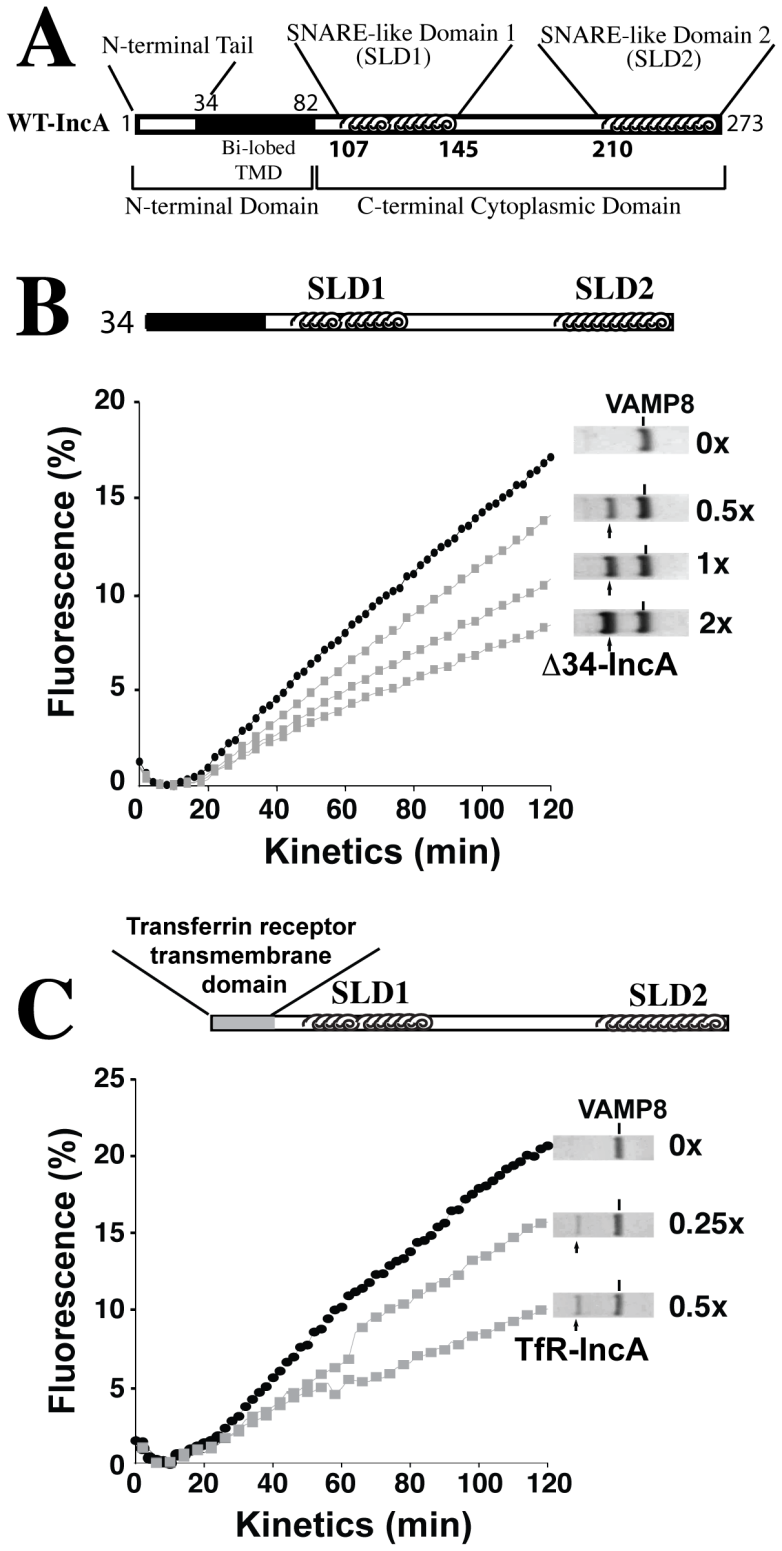
The N-terminal tail region and transmembrane domain of IncA are dispensible for the inhibitory function of IncA. (A) Schematic of full-length wildtype IncA. The N-terminal tail encompasses the first 34 amino acids. The transmembrane domain is depicted by the black area (amino acids 35–82). SNARE-like domain1 (SLD1) is located between amino-acids 107 and 145; SLD2 between amino-acids 210 and 273. (B) 45 µL of t-SNARE liposome reconstituted with Stx7, Stx8, Vti1b were mixed with 5 µL of v-SNARE liposome reconstituted with VAMP8 and an increasing concentration of IncA. NBD fluorescence was measured every 2 min for 2 hrs at 37°C. 10 µL of n-Dodecyl-β-D-maltoside (2.5% w/v stock concentration) was added to the reaction and data were normalized to the 100% fluorescence value as described [24]. Protein gel lanes show co-reconstitution of Δ34-IncA with VAMP8 on v-SNARE liposomes. Values next to protein gel lanes refer to the ratio of IncA mutant to VAMP8 for that particular fusion curve. (C) Fusions were performed as in (B) except that the N-terminal tail region and TMD of IncA were replaced with the TMD of the transferrin receptor (TfR) before reconstituting increasing concentration of this chimeric protein with VAMP8. For (B) and (C), black circles represent fusion in the absence of IncA mutant and gray squares represent fusion in the presence of increasing concentrations of IncA mutant relative to VAMP8. Results are representative of at least four independent experiments.

Since IncA has an optimal inhibitory function when inserted with the v-SNARE (Figure 1D), we next tested the function of each IncA domain (Fig. 2A) using this configuration. Previously, we have demonstrated a role for SLD1 in the inhibition of SNARE-mediated liposome fusion [20]. The observation that an IncA mutant lacking SLD2 was still capable of inhibiting fusion suggested that SLD1 was sufficient to confer inhibitory ability. Here, we determined whether SLD1 requires the input of other domains–namely, the N-terminal tail (amino acids 1–34) and/or the transmembrane domain (TMD)–to successfully inhibit membrane fusion. To test for the importance of this domain, we generated an IncA mutant lacking the N-terminal tail region (Δ34-IncA).

When Δ34-IncA is co-reconstituted with VAMP8, fusion is still significantly inhibited (Fig. 2B). Furthermore, we found that increasing the ratio of Δ34-IncA to VAMP8 on liposomes consistently leads to more pronounced inhibition. At a 1∶2 molar ratio, normalized NBD fluorescence decreases from ∼17% for liposomes not containing Δ34-IncA to ∼14% (an 18% overall decrease) (Fig. 2B). At a 1∶1 ratio, normalized fluorescence decreases by about 35%, and for a 2∶1 ratio, there is a 53% decrease in signal. The correlation between increasing ratios of Δ34-IncA:VAMP8 and lower levels of NBD fluorescence suggests that the observed inhibition is specific for Δ34-IncA and is consistent with previous observations for wildtype IncA [20]. In all, these data show that the N-terminal tail region is dispensable for IncA’s inhibitory function.

We then investigated whether the TMD of IncA contributes to inhibition. Interactions between cognate SNAREs are stabilized by the participation of their TMDs [27], [28]. We reasoned that the bi-lobed TMD of IncA may promote inhibition of membrane fusion by directing IncA to sites of SNARE complex formation or, more generally, to areas of membrane occupied by SNARE proteins via hydrophobic interactions. To address this possibility, we purified a mutant of IncA in which the N-terminal tail region and TMD were replaced by a non-SNARE TMD, namely that of the transferrin receptor. DNA sequencing of the transferrin receptor-IncA chimera reveals a TMD of ∼24 amino acids compared to ∼40 amino acids for IncA, making the TMD considerably smaller (data not shown).

Similar to Δ34-IncA, TfR-IncA inhibits lipid mixing in a dose-dependent fashion (Fig. 2C). At a 1∶4 ratio of TfR-IncA:VAMP8, NBD fluorescence decreases by ∼25%, and for a 1∶2 ratio, fluorescence decreases by about 50%. Taken together, these results suggest that neither the N-terminal tail region nor the hydrophobic TMD of IncA is necessary for the inhibition of late endocytic SNARE-mediated liposome fusion. This confirms that the inhibitory ability of IncA is confined to the C-terminal cytoplasmic domain containing the two putative coiled-coil domains.

### SLD2 Inhibits Late Endocytic SNARE-Mediated Fusion Independently of SLD1

The C-terminal cytoplasmic domain of IncA encodes two SNARE-like domains, SLD1 and SLD2 [20]. We hypothesized that SLD2 was also able to inhibit liposome fusion based on several observations. First, in addition to showing a high propensity towards coiled-coil formation, SLD2 contains a glutamine residue that aligns with other glutamine residues conserved in the 0-layer of a variety of SNARE proteins including Syntaxins 6, 7, 8, and 16 [22]. Secondly, mutating this residue to an arginine diminished IncA binding to VAMP8 in an *in vivo* pulldown assay [22].

To determine whether SLD2 was independently capable of inhibiting fusion, we first introduced point mutations into SLD1 to abolish its functionality. We chose to inactivate the function of SLD1 using point mutations instead of truncations in order to keep the overall organization of the protein intact. In particular, this strategy allows the location of SLD2 relative to the transmembrane domain to be conserved.

**Figure 3:**
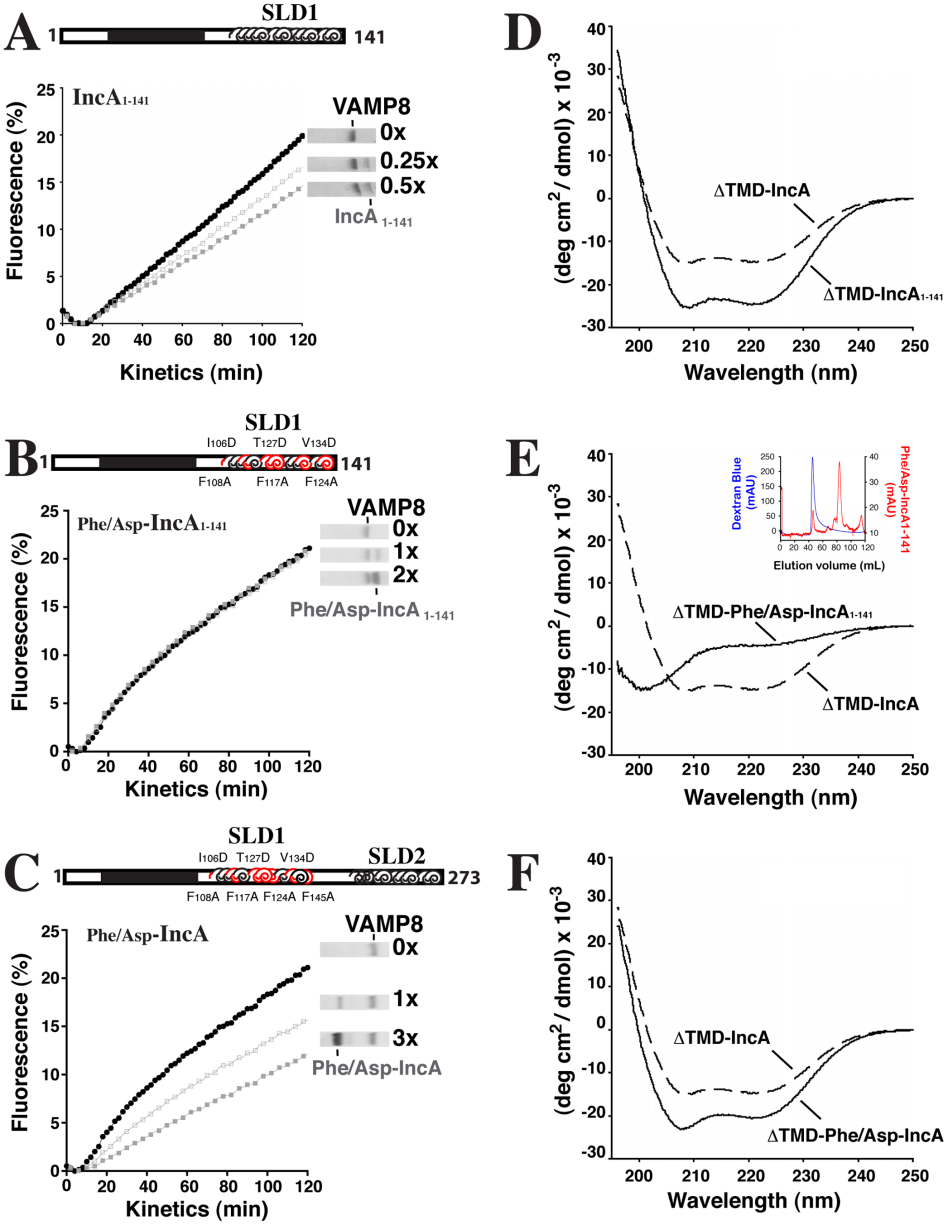
SLD2 inhibits late endocytic SNARE-mediated fusion independently of SLD1. (A) SLD1 is sufficient for SNARE inhibition. Increasing concentrations of IncA_1–141_ were reconstituted in v-SNARE liposomes with VAMP8 and mixed with t-SNARE liposomes in which Stx7, Stx8, and Vti1b were reconstituted. Fusion rates were calculated as described in the legend of Figure 2B. Protein gel lanes show co-reconstitution of VAMP8 and IncA_1–141_. (B) Phenylalanine and Aspartic acid mutations inactivate SLD1. Increasing concentrations of Phe/Asp-IncA_1–141_ were reconstituted in v-SNARE liposomes with VAMP8. v-SNARE liposomes were mixed with t-SNARE liposomes and data analyzed as in (A). (C) SLD2 independently inhibits SNARE-mediated membrane fusion. Increasing concentrations of Phe/Asp-IncA were reconstituted in v-SNARE liposomes with VAMP8 and v-SNARE liposomes were mixed with t-SNARE liposomes. Data were analyzed as in (A). In A, B, and C, black circles represent fusion in the absence of IncA and gray squares represent fusion using liposomes containing IncA construct. Results are representative of at least four independent experiments. (D, E, F) Far-UV CD wavelength scans of IncA constructs. Protein was dissolved in CD Buffer (200 mM NaF, 20 mM Tris, 10% glycerol, 1 mM DTT, pH7.4) to a final concentration of 10 µM and far-UV scans were taken in a 1 mm pathlength quartz cuvette (Starna) at 20°C. Data are the averages of five scans. For comparison, the CD signal of ΔTMD-IncA (which contains wildtype SLD1 and SLD2) is shown as a dashed curve. (D) SLD1 displays an α-helical structure. ΔTMD-IncA_1–141_ was dissolved in CD Buffer and analyzed as above. (E) Phenylalanine and Aspartic acid mutations interfere with the α-helical structure of SLD1. ΔTMD-Phe/Asp-IncA_1–141_ was dissolved in CD Buffer and analyzed as in (D). The loss of structure cannot be attributed to aggregation of the protein. Inset shows elution profile of protein (red line) superimposed on elution profile of blue dextran (blue line) to determine void volume. The protein elutes far from the blue dextran indicating a soluble species of protein. (F) Phe/Asp-IncA displays an α-helical structure. ΔTMD-Phe/Asp-IncA was dissolved in CD Buffer and analyzed as in (D).

The primary sequence of SLD1 reveals a putative 3–4 heptad repeat of the form *a-b-c-d-e-f-g* where the “*a*” and “*d*” residues are hydrophobic. We mutated the amino acids isoleucine, valine, and threonine located in the “*d*” positions in SLD1 to aspartates because β-branched amino acids in this position have been shown to stabilize coiled-coil formation [29], [30]. Furthermore, we mutated the phenylalanines in SLD1 to alanines. Phenylalanines are prevalent in SNARE motifs and have been shown to stabilize coiled-coil membrane proteins [31]. Mutations are depicted as red coils in Figure 3 (B and C).

Truncated IncA mutants containing wildtype SLD1 (IncA_1–141_) inhibit fusion approximately 25% at IncA:VAMP8 ratios of 1∶2 (Fig. 3A), consistent with our previous results [20]. When the above mutations were introduced into this region of IncA (Phe/Asp-IncA_1–141_), this mutant became incapable of inhibiting late endocytic SNARE-mediated fusion even at IncA:VAMP8 ratios of 2∶1, demonstrating that this series of mutations was effective to completely inactivate SLD1 (Fig. 3B). When these mutations were introduced into the full-length IncA protein (Phe/Asp-IncA) in which SLD2 is still active, robust inhibition is restored (Fig. 3C). This suggests that SLD2 has an inhibitory function that can act independently of SLD1.

The considerable loss in activity of IncA could be explained by a concomitant loss of structure in the Phe/Asp-IncA_1–141_ mutant. To test this possibility, we purified peptides corresponding to the SNARE-like domain(s) of wildtype IncA, IncA_1–141_, Phe/Asp-IncA_1–141_, and Phe/Asp-IncA and secondary structure was analyzed using circular dichroism (CD) spectroscopy. These constructs lack the transmembrane domain and the N-terminal tail region and are denoted as “ΔTMD.” ΔTMD-IncA and ΔTMD-IncA_1–141_ show marked α-helical content as evidenced by the double minima around 208 nm and 222 nm (Fig. 3D). Interestingly, ΔTMD-Phe/Asp-IncA_1–141_ shows no signs of secondary structure, indicating that these mutations abolish α-helicity in this region (Fig. 3E). The lack of secondary structure is not due to aggregation of the peptide because the protein elutes far from the void volume on a size exclusion column (Fig. 3E, inset). Interestingly, the CD spectra of ΔTMD-Phe/Asp-IncA reveals strong α-helical content, suggesting SLD2 may fold independently of SLD1 (Fig. 3F). Together, these data suggest that IncA contains two CCDs that inhibit SNARE-mediated fusion independently of one another. The loss of α-helicity correlates with a loss of inhibitory capacity, however additional experiments will be necessary to address a possible link between α-helicity and inhibitory activity. These observations are consistent with IncA being an inhibitory SNARE-like protein and establish for the first time the inhibitory role of SLD2.

### Both SNARE-like Domains are Required to Induce Homotypic Fusion of the Inclusions in Host Cells

In addition to inhibiting fusion, IncA is involved in the homotypic fusion of the inclusions via oligomerization [3] The process of homotypic fusion is a critical step in the pathogenicity of *C. trachomatis*. Failure of the inclusions to fuse results in decreased bacterial loads and, in infected individuals, subclinical disease outcomes [4], [32]. IncA is a key player in this event because microinjection of blocking antibodies to IncA or inhibition of type III secretion results in the formation of nonfusogenic *C. trachomatis* inclusions [32]–[34]. The domains of IncA that play significant roles in this process are not known. Next, we determined whether the SNARE-like domains of IncA also play a role in homotypic fusion.

**Figure 4:**
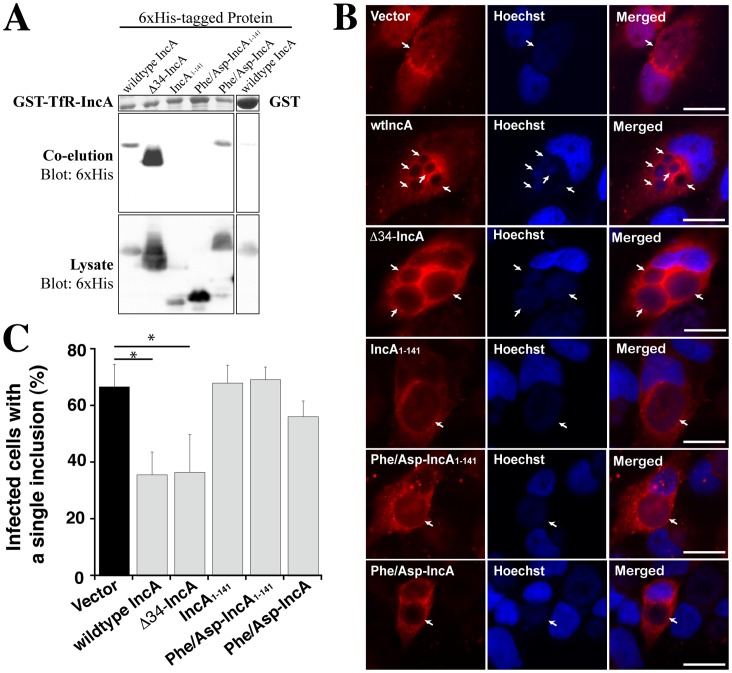
SLD2 is required for oligomerization but both SNARE-like domains are necessary for homotypic fusion. (A) SLD2-containing IncA mutants co-elute with GST-TfR-IncA. 6xHis-tagged IncA mutants were co-expressed with GST-TfR-IncA in BL21(DE3) *E. coli*, and GST-containing complexes were purified over glutathione beads. Co-eluted IncA mutants were detected by western blot using anti-6xHis antibody. GST-TfR-IncA was visualized by Coomassie Blue staining. IncA mutants that contain SLD2 co-precipitated while the two truncated mutants lacking SLD2 did not. GST control shows basal levels of wildtype 6x-His-IncA binding. Results shown are typical of five independent experiments. (B) Transfection with wildtype- or Δ34-IncA leads to nonfusogenic inclusions. HeLa cells were transfected with plasmids expressing the indicated DsRed-IncA mutant or vector control (pDsRed-monomer-C1) and subsequently infected with *C. trachomatis* L2 for 24 hr. The location of each DsRed-IncA construct is shown on the left (red), while the inclusions are shown in the middle (blue, Hoechst staining). The right column shows the overlay. Arrows denote inclusions. Note the multiple inclusions in wildtype-IncA and Δ34-IncA transfected cells compared to the cells transfected with other IncA constructs. Scale bars represent 10 µm. Images are typical of four independent experiments. (C) Expression of either wildtype or Δ34-IncA inhibits subsequent inclusion development in HeLa cells. The number of inclusions/cell was determined by fluorescence microscopy. More than fifty infected cells per replicate per transfection were assessed for multiple inclusions. The number of infected cells carrying a single inclusion was divided by the total number of infected cells and expressed as a percent. Data are averages of four independent experiments. Error bars represent one standard deviation. Asterisks denote a significant difference compared to control-transfected cells (p<0.05).

We first performed co-elution experiments to identify the domains that contribute to the multimerization of IncA. GST-tagged TfR-IncA was co-expressed with 6xHis-tagged IncA mutants in BL21(DE3) *E. coli*. Using the TfR-IncA chimera allowed us to determine the contribution of the IncA TMD to multimerization. GST-tagged complexes in the BL21(DE3) lysate were affinity purified using glutathione agarose beads, and the resulting complexes were analyzed by SDS-PAGE (Fig. 4A, co-elution blot). As a control, a plasmid expressing only GST was co-transformed with the plasmid expressing 6xHis-tagged wildtype IncA (Fig. 4A, right columns). A striking pattern emerged in our co-elution experiments in that only those 6xHis-tagged IncA mutants that contained SLD2 co-purify with GST-TfR-IncA (Fig. 4A). Wildtype, Δ34-IncA, and Phe/Asp-IncA all co-purify while IncA_1–141_ whereas Phe/Asp-IncA_1–141_ does not. The N-terminal tail region and the native transmembrane domain of IncA are seemingly both dispensable for oligomerization as Δ34-IncA (which lacks the N-terminal tail region) co-purifies with GST-TfR-IncA (lacking both the N-terminal tail region and the TMD). The lack of binding for both IncA_1–141_ and Phe/Asp-IncA_1–141_ is not due to their low expression level as shown in the loading control (Fig. 4A, lysate blot).

Transfection of HeLa cells with wildtype IncA leads to growth defects of developing inclusions due to the interaction of transgenic IncA with endogenous IncA on the inclusion [35]–[37]. The mechanism for this phenotype has yet to be elucidated, but is believed to be the result of inhibition of homotypic fusion between two or more inclusions in the same cell [3], [34], [36]. Therefore, IncA mutants that do not bind endogenous IncA (i.e. that do not contain SLD2) should not lead to developmental defects. To test this hypothesis, we transgenically expressed wildtype IncA, Δ34-IncA, IncA_1–141_, Phe/Asp-IncA_1–141_, and Phe/Asp-IncA fused to the monomeric form of DsRed in HeLa cells 24 h prior to infection with *C. trachomatis* strain L2 and monitored changes in inclusion morphology associated with each mutant. For this experiment, we considered only those cells that were transfected (DsRed-positive). We found that the overall frequency of infection (ratio of infected cells to total cells) did not vary significantly (data not shown). However, we observed marked differences in inclusion morphology between empty-vector control and DsRed-IncA-expressing HeLa cells (Fig. 4B). DsRed staining in control cells was diffuse and single large inclusions are highly visible (Fig. 4B, first row). Transfection with DsRed-IncA (Fig. 4B, second row) leads to the formation of multiple inclusions that are reminiscent of nonfusogenic inclusions described previously (p = 0.001, Fig. 4C) [3], [33], [34]. Interestingly, transfection with DsRed-Δ34-IncA also leads to non-fusogenic inclusions (Fig. 4B, third row and Fig. 4C p = 0.008), suggesting that Δ34-IncA is functional and that the N-terminal tail does not play a role in homotypic fusion. As expected from our co-elution data, DsRed-IncA_1–141_ and DsRed-Phe/Asp-IncA_1–141 _do not have any effect on inclusion development compared with vector control (Fig. 4B, fourth and fifth rows, respectively and Fig. 4C). Surprisingly, transfection with DsRed-Phe/Asp-IncA, which contains SLD2 (but in which SLD1 is inactivated), fails to inhibit inclusion development (Fig. 4C, *p* = 0.072). Inclusions appeared normal as in the vector control, suggesting that Phe/Asp-IncA lost its activity in homotypic fusion (Fig. 4B, sixth row). We conclude that, although SLD2 is necessary and sufficient for oligomerization *in vitro*, under physiologic conditions, it requires SLD1 to be fully functional and promote homotypic fusion of inclusions.

## Discussion

*Chlamydia trachomatis* is one of the most skilled pathogens in co-opting host fusion, able to inhibit host pathways while inducing new fusion events. The *Chlamydia* SNARE-like protein IncA appears to play a major role in both of these processes. During infection, IncA is involved in both the inhibition of host endocytic SNARE-mediated membrane fusion [20] and in the induction of inclusion homotypic fusion [3], [33]. How IncA establishes a delicate balance between *blocking* endosomal/lysosomal membrane fusion events and *activating* new fusion events between inclusions is unknown, and, despite its importance in manipulating membrane fusion, a detailed mechanism of IncA function remains unknown. In the present study, we first established that IncA must be located on the same membrane than the target SNAREs to exert its inhibitory function (Fig. 1). This result is consistent with the restricted disturbance of membrane fusion by the inclusion, which leaves other vesicle trafficking events intact. In fact, *Chlamydia* does not affect vesicular trafficking in the rest of the cell as the general endocytic pathway in infected cells remains functional [11].

IncA is organized in distinctive domains (see Fig. 2A). The N-terminal region of the protein encompasses a tail region (residues 1–34) and a bilobed transmembrane domain (TMD), for which no specific function is known. The tail region reveals no obvious signature of α-helices or β-sheets based on secondary structure prediction algorithms, yet it is facing the host cytosol where it has the potential to interact with host proteins and manipulate membrane fusion [21]. The transmembrane domain (TMD) has unique characteristics. At ∼40 amino acids, it is about twice as long as that of a typical single pass transmembrane protein, and has been termed “bi-lobed” [38]. For comparison, the SNARE proteins used in this study Stx7, Stx8, Vti1b, and VAMP8 each have TMDs of between 20 and 23 residues. In the present study, domain swap/deletion experiments conclusively show that both these domains are dispensable for IncA to inhibit SNARE complexes since their removal did not abrogate its function (Fig. 2C). Furthermore, the deletion of the N-terminal tail does not prevent IncA from multimerizing (Fig. 4A), and transgenic expression of Δ34-IncA inhibited subsequent inclusion development. Altogether, these results demonstrate that the N-terminal domain containing the N-terminal tail and the transmembrane domain are not functionally involved in membrane fusion. However, it has been previously shown that the N-terminal domain functions in the targeting of the protein to the inclusion membrane through the type 3 secretory system [39]. Thus, it seems that the function of the N-terminus of IncA is restricted to protein translocation to the inclusion surface.

As for the cytosolic C-terminal domain of IncA, it contains two SNARE-like domains, SLD1 and SLD2. Previously, we have shown that the N-terminal SNARE-like domain SLD1 is sufficient to inhibit endocytic SNARE-mediated membrane fusion [20]. The molecular dissection of IncA presented in this study now reveals a role for the C-terminal SNARE-like domain SLD2. By targeting key residues important for α-helicity and coiled-coil integrity in SLD1, we first inactivated this domain, thus only leaving SLD2 active (Fig. 3). Using an *in vitro* fusion assay, we then observed that SLD2 independently contributes to the inhibition of SNARE-mediated membrane fusion. Intriguingly, the loss of inhibitory function of SLD1 correlates with the loss of secondary structure as observed by far-UV circular dichroism analysis. Although more experiments will be required to functionally validate this observation, it is consistent with the hypothesis that SNARE-like domains require α-helical structure in order to be active.

We can speculate on a functional mechanism for each IncA SLD based on studies of the eukaryotic membrane fusion system. One possibility is that IncA binds the SNAREs and functions like inhibitory SNAREs, or i-SNAREs. i-SNAREs fine-tune SNARE-mediated fusion by inhibiting constitutive SNARE complex formation and membrane fusion [40]. Varlamov et al. showed that the yeast Golgi SNARE complex composed of Sed5, Sec22, Bos1, and Bet1, is inhibited by the presence of non-cognate SNAREs Gos1, Tlg1 and Sft1 on the liposome [40]. Moreover, this effect is dose-dependent because adding more Bos1 to the t-SNARE liposome suppressed the inhibitory phenotype suggesting that the i-SNAREs were forming nonfusogenic pseudo-SNARE complexes [40]. IncA could also form nonfusogenic pseudo-SNAREs by competing with one or all of the t-SNAREs (Stx7, Stx8, Vti1b) to bind to VAMP8. Computational modeling and *in vitro* pulldown data support this scenario [22]. Subtil and co-workers showed that a tetrameric complex consisting of three SLD2 domains and one VAMP8 molecule would likely be structurally similar to a bona-fide SNARE four-helix bundle [22]. Alternatively, the formation of non-functional SNARE complexes is known to occur in other biological contexts and IncA could function in a different way. The proteins of the complexin family, for example, insert themselves in an antiparallel orientation into SNARE complexes and create alternative coiled-coils that effectively arrest SNAREs in a non-fusogenic state [41]–[43]. It is tempting to speculate that alternative coiled-coil formation evolved as an efficient strategy to block membrane fusion and control vesicle traffic and that intracellular pathogens may have acquired similar systems to thwart host defenses. Structural studies will be required to discriminate between both of these possibilities.

The previously noted correlation between *inca* expression and homotypic fusion, combined with the presence of coiled-coil domains resembling SNARE motifs, led us to hypothesize a role for these domains in activating fusion. To test this hypothesis, we first assessed the IncA mutants for their ability to oligomerize, since IncA oligomerization appears to be part of the fusion process [3]. Using a co-elution strategy, we found that only those IncA mutants containing SLD2 co-eluted with GST-TfR-IncA that encodes an intact C-terminal cytoplasmic domain (Fig. 4A). Surprisingly, we found that *both* SLDs are required to efficiently interfere with homotypic fusion in a cellular assay, suggesting that both domains are involved in the formation of a fusogenic coiled-coil bundle [22]. These results are consistent with previous yeast two-hybrid and microscopy assays, which determined that the C-terminal cytoplasmic domain is necessary to promote IncA oligomerization and fusion of multiple inclusions in the same cell [3]. Similar to the fusogenic SNARE four-helix bundle [44], [45], our results suggest that IncA may require both SLDs in order to promote homotypic fusion.

While IncA is required for homotypic fusion, it is likely not sufficient for fusion because species that do not fuse inclusions such as *Chlamydophila psittaci*, contain homologous *inca* genes [46]. It should also be noted that amino acid sequence conservation between Inc proteins of different species is relatively low [38]. Therefore, sequence orthology may not imply functional homology, and the non-fusogenic phenotype could be due to genetic variation in *inca*. More experiments are needed to determine the exact mechanism of inclusion fusion.

Altogether, these results imply that the function of each SLD may be context dependent. Either domain is capable of inhibiting late endosome/lysosome fusion to shield the inclusion from degradation (Fig. 3), but both domains appear to be required to promote homotypic interaction between inclusions (Fig. 4). It is conceivable that *C. trachomatis* evolved redundant CCDs capable of inhibiting SNARE fusion as a way to protect itself from deleterious mutations to one or both domains over time. The ability of IncA to promote homotypic interaction between *C. trachomatis* inclusions could have arisen later in evolutionary time partly as a consequence of the two CCDs. Additional molecular components are likely to be involved in the homotypic fusion process since all *Chlamydia* IncA homologues present two CCDs, yet, only IncA expressed by *C. trachomatis* is implicated in homotypic fusion [3].

## Future Directions

If SNARE-like proteins are such an important and efficient system to corrupt host SNAREs and manipulate membrane fusion, could they be used by other intracellular bacteria? The intracellular bacterial pathogen *Mycobacterium tuberculosis* was responsible for 1.4 million deaths in 2011 (World Health Organization). Like *Chlamydia,* one key aspect of the pathogenicity of mycobacteria is interference with lysosomal fusion. Although some strategies that mycobacteria use to block phagosomal maturation are known, their impact on the SNARE fusion machinery is understudied. Do they use inhibitory SNARE-like proteins and co-opt host SNAREs to establish and protect their infectious compartment? The same question applies to many more intracellular bacteria, including *Salmonella typhi*, *Brucella abortus*, *Legionella pneumophila*, all significant health and economic burdens. Ultimately, the discovery that a variety of intracellular bacteria utilize SNARE-like proteins to protect their infectious compartment implies evolutionary conservation, and could lead to a major therapeutic effort in this direction.

## Supporting Information

Table S1 **List of DNA primers used in this study. Primer designations (FO#) and their sequences (5′-to 3′) are shown in the table.**
(PDF)
