# Supplementary material for: Two Coiled-Coil Domains of Chlamydia trachomatis IncA Affect Membrane Fusion Events during Infection
Source: PLoS One. 2013 Jul 23;8(7):e69769. doi: 10.1371/journal.pone.0069769 (PMC3720611; doi:10.1371/journal.pone.0069769)
Supplement: Table S1 — List of DNA primers used in this study. Primer designations (FO#) and their sequences (5′-to 3′) are shown in the table. (PDF) [file pone.0069769.s001.pdf]

List of DNA Primers Used in Study

Primer designation:	Sequence (5'-3')
FO124	ATAAGAATTCTATGACAACGCCTACTCTAAT
FO125	TTATGGATCCCTAGGAGCTTTTTGTAGAGGG
FO162	GATGGATCCCTAGTCTTTAGATGTCGTTGCAAAT
FO136	CGCGGATCCATGATAGCAGCCATTGCCTCCCTAATT
FO137	GCGAATTCCTAGGAGCTTTTTGTAGAGGG
FO401	TGGGTCTCTAAAAGAAGATAATTTCATGCTGAGCGTTCT
FO402	AAAAATCTTGAGAATCAGCAGAGAGGTCTTTAGAATCCGTTGCAAATTCTTTAGA
FO414	GAAGATAATGCCATGCTGAGCGTTCTACAGAAAGAAGCTCTTCATTTATCTAAAGAAGCTGCAACGGATTCTAAAG
FO415	ATAGTTATCTCTAGCTCCTTGCAAACAAGAATAAGCATCTTGAGAATC
FO416	GAATTCCTAAGAATAAGCATCTTGAGAATC
FO399	GGGAATTCCATATGACCTCATTAGCAGGGAATGGG
FO400	CGCGGATCCTCACTCATCCAAAAGAGATTC
FO441	CCGGAATTCTATAGCAGCCATTGCCTCCC
